# Hydrogel assisted synthesis of gold nanoparticles with enhanced microbicidal and in vivo wound healing potential

**DOI:** 10.1038/s41598-022-10495-3

**Published:** 2022-04-21

**Authors:** Zahra Batool, Gulzar Muhammad, Muhammad Mudassir Iqbal, Muhammad Shahbaz Aslam, Muhammad Arshad Raza, Noreen Sajjad, Muhammad Abdullah, Naeem Akhtar, Asad Syed, Abdallah M. Elgorban, Salim S. Al-Rejaie, Zahid Shafiq

**Affiliations:** 1 Institute of Chemical Sciences, Bahauddin Zakariya University, Multan, 60800 Pakistan; 2 Department of Chemistry, GC University Lahore, Lahore, Pakistan; 3 Department of Chemistry, Superior University, Lahore, Pakistan; 4 School of Biochemistry and Biotechnology, University of the Punjab, Lahore, 54000 Pakistan; 5 Department of Chemistry, University of Lahore, Lahore, Pakistan; 6 Department of Chemistry, University of Massachusetts Amherst, 710 North Pleasant Street, Amherst, MA 01003 USA; 7 Interdisciplinary Research Centre in Biomedical Materials (IRCBM), COMSATS University Islamabad, Lahore Campus, Lahore, 54000 Pakistan; 8 Department of Botany and Microbiology, College of Science, King Saud University, P.O. 2455, Riyadh, 11451 Saudi Arabia; 9 Department of Pharmacology and Toxicology, College of Pharmacy, King Saud University, P.O.Box 55760, Riyadh, 11451 Saudi Arabia

**Keywords:** Experimental organisms, Analytical chemistry, Biochemistry, Materials chemistry

## Abstract

The present study reports a hydrogel-based sunlight-assisted synthesis of gold nanoparticles (Au NPs) with enhanced antimicrobial and wound healing potential. The hydrogel extracted from the seeds of *Cydonia oblonga* was used as a reducing and capping agent to synthesize Au NPs for the first time. The as-synthesized Au NPs were characterized for an average size, shape, surface functionalization, antimicrobial, and wound healing capabilities. The cubic and rectangular-shaped Au NPs with an average edge length of 74 ± 4.57 nm depicted a characteristic surface plasmon resonance band at 560 nm. The hydrogel-based Au NPs inhibited the growth of microorganisms in zones with 12 mm diameter. In-vitro experiments showed that a minimum inhibitory concentration of Au NPs (16 µg/mL) was sufficient to mimic the 95% growth of pathogenic microorganisms in 24 h. In vivo treatment of wounds with Au NPs in murine models revealed a 99% wound closure within 5 days. Quantitative PCR analysis performed to decipher the role of Au NPs in enhanced wound healing showed an increase in the expression levels of NANOG and CD-34 proteins.

## Introduction

Despite tremendous advancement, versatile applications of nanoparticles (NPs) still fascinate researchers worldwide to develop new synthetic methodologies. The medical uses of NPs include but are not limited to eye medication, dental regimen, catheter coating, wound dressing, antimicrobial filters, and sanitizing medical devices^1,2^. The higher microbicidal characteristics of NPs owe to the fact that they more conveniently penetrate the cell membranes and cell walls of pathogens compared to typical anti-fungal and anti-bacterial drugs^3,4^. For example, incorporation of gold and selenium-based NPs into the nanofibers significantly increases the antimicrobial properties of the polymer-based wound dressing^5,6^. Similarly, Au-Se hybrid NPs implanted into cellulose-based polymeric nanofibrous wound dressings have been reported to increase their antimicrobial activity^7^. Silver and gold-based NPs have also been employed as carriers for blackberry extract to treat cisplatin-induced cardiotoxicity^8^. Carvacrol-based nanoemulsion efficiently attenuating the neurodegenerative disorders in diabetes is another example of biomedical application of NPs^9^.

Recently, several chemical, physical, and green methods for the synthesis of NPs have been reported^10–12^. The non-toxic nature of Au NPs compared to other metallic NPs such as Ag and Pt has been well-established^13^. Turkevich et al*.,* have reported the most commonly employed method for the synthesis of Au NPs using citrate as a reducing agent^14^. Au NPs can also be synthesized by the reduction of Au(III) salts using gallic acid, hydrogen peroxide, and hydrazine as reducing agents^15,16^. Some of these reducing agents such as citric acid, oleyl amine, sodium borohydride (NaBH_4_), trioctyl-phosphine, hexadecyltrimethylammonium bromide, and polyethylene glycol are considered toxic, combustible, and perilous to the environment^17,18^. Thus, there is a dire demand to develop clean, biocompatible, and eco-friendly methodologies for the synthesis of NPs^19^.

The green methods for the synthesis of NPs using bacteria, fungi, actinomycetes, algae, and plants are considered eco-friendly and economical^20,21^. These methods, which are also known as biosyntheses, typically utilize the natural products and hydrogels found in living organisms as reducing and capping agents^22^. The availability, biodegradability, and biocompatibility are significant characteristics of hydrogels that draw the attention of scientists to use them as capping and reducing agents for the synthesis of NPs^23–25^. Hydrogels possess 3D hydrophilic structures with extensive cross-linking^26^ and absorb a huge quantity of aqueous solutions due to the presence of certain functional groups such as −OH, −COOH, −SO_3_H, and −CONH_2_^23^.

Hydrogel has been used in the preparation of Ag NPs, however, until now, there has been no report on the preparation of Au NPs^27^. The current research presents a sunlight-assisted green synthesis of Au NPs using hydrogel extracted from *C. oblonga* seeds which act as a stabilizing and reducing agent. The as-prepared Au NPs were characterized using scanning electron microscopy (SEM), UV–Vis and Raman spectroscopies, energy-dispersive X-ray (EDX), and dynamic light scattering (DLS). The synthesized NPs were evaluated for the antimicrobial activity against different bacterial (*B. subtilis*, *B. simplex*, *S. aureus*, *P. aeruginosa*, and *E. coli*), and fungal strains (*P. notatum* and *A. niger*)*.* The formation of zones of inhibition, minimum inhibition concentration (MIC) values, and the effect of Au NPs on wound healing in the murine models was thoroughly investigated.

## Method of synthesis

### Materials

The plant (*C. oblonga*) seeds were purchased from Punjab Seed Corporation, Pakistan, and were verified by the Department of Botany, Government College University, Lahore, Pakistan. The seeds were cleaned and stored at room temperature. The gold precursor, hydrogen tetrachloroaurate (III) (HAuCl_4_, 99.98%) was bought from Merck, Darmstadt, Germany. All the solutions were prepared in deionized water. CYBER green real-time PCR kit for quantitative analysis of biomarkers involved in wound healing was purchased from Thermo Fisher Scientific (USA). All experiments involving the seeds (seed collection, authentication, and use in research) follow the guidelines of IUCN Council, Gland, Switzerland, and CITES of Wild Fauna and Flora, Geneva, Switzerland.

### Hydrogel mediated synthesis of Au NPs

Hydrogels are widely employed for the green synthesis of nanoparticles. The hydrogels adsorb plenty of water and metal ions which can easily penetrate through the polymeric matrix of the hydrogel^28^. Initially, the plant seeds (200 g) were soaked in 1.0 L water at room temperature for 20 min. The hydrogel extracted from the seeds was separated using a cotton muslin cloth. The isolated hydrogel was desiccated at 60–65 °C for 4–5 h in a hot air oven and then ground to a fine powder. The suspension of hydrogel (1.0% w/v) was prepared by suspending hydrogel powder (1.0 g) in 100 mL deionized water. The solution (100 mmol) of the gold precursor was prepared by dissolving 0.393 g of HAuCl_4_ in deionized water (10.0 mL). The freshly prepared hydrogel suspension (10 mL) was then mixed with HAuCl_4_ solution (10 mL, 100 mmol) and stirred at room temperature. The progress of the reaction was monitored by colour variation from yellow to reddish-brown and the localized surface plasmon resonance (LSPR) absorption bands for 2 h using a UV–Vis spectrophotometer.

### Characterization of Au NPs

Properties of NPs depend upon various features like average particle size, size distribution, the shape of particles, surface functionalization, and tendency to agglomerate. The as-synthesized Au NPs were investigated by Raman and FTIR spectroscopy, TEM, and DLS measurements. The reduction of gold ions to Au NPs was monitored by measuring absorption at regular intervals, i.e. 30, 60, 90, and 120 min with 800–200 nm wavelength range using Agilent Cary 60 spectrophotometer. Raman spectrum of Au NPs was recorded using inVia Raman (RENISHAW UK) with 5% laser power, laser beam excitation (488 nm), and 1800 lines/mm of grating with 20× objective lens and 10 s of laser exposure time at 25 °C.

Surface morphology of Au NPs was investigated by depositing a drop of synthesized nanoparticles on the carbon-coated copper grid using a microscope (FEI Cs-corrected Titan 80–300 kV microscope with an accelerating voltage of 200 kV.

Dynamic light scattering (BT-90 NANO PSA Bettersize) was used for determining the particle size distribution and the nature of the particle's motion in the medium. The DLS system is equipped with a 635 nm He–Ne laser and an avalanche photodiode detector configured to collect backscattered light at 90°. The sample was held at 30 ℃ by a temperature-controlled sample holder to equilibrate for 60 s before each measurement. All DLS data were collected and analyzed using 2.0-CONTIN analysis mode. All reported mean particle hydrodynamic diameters (D_H_) are calculated from intensity-based particle size distributions. Each sample was analyzed in triplicate to calculate an average and standard deviation. Rigaku MiniFlex 600-C was used for X-Ray Diffraction. It has a Cu (Kα) based X-Ray tube operated at 600 W with a graphite monochromator and silicon strip detector.

### Anti-fungal and anti-bacterial activity of Au NPs

The microbicidal and fungicidal activities of Au NPs were analyzed against bacterial strains *B. simplex*, *B. subtilis*, *E. coli*, *P. aeruginosa*, and *S. aureus*, and fungal strains *A. niger* and *P. notatum.* Luria Bertani (LB) medium is nutrient-rich and is generally recommended to grow bacteria for various experiments. The bacterial strains were grown in LB medium (Thermo Fisher Scientific, MA, USA) with agitation at 150 rpm in an orbital shaker at 37 °C, while the growth of bacteria on LB agar plates doesn't require agitation. The fungal strains were grown on sterile potato dextrose agar medium (Thermo Fisher Scientific, MA, USA) at 25 °C for 7 days.

A simple disc diffusion method on agar plates was employed to analyze the formation of the zone of inhibition. Initially, purified bacterial strains were grown in a sterile growth medium (10 mL) for 12 h at 150 rpm and 37 °C. This bacterial culture (100 µL) with OD_600_ was spread on sterile LB agar plates. Then sterile filter paper discs (Whatman no. 1, 6 mm) were dipped in a solution of Au NPs (100 µL) and placed on sterile LB agar plates expanded with bacterial cultures. This was followed by incubation of these plates at 37 °C for 12–24 h. Ampicillin and sterile water were used as positive and negative controls, respectively. The process was imitated with five bacterial strains. The same procedure was adapted for fungal strains grown on potato dextrose agar medium. The graphs were plotted between the zone of inhibition and the microbial strains.

For the analysis of MIC value, the bacteria were grown in liquid broth in the presence of different concentrations of Au NPs. Initially, the bacterial cultures were grown on a 3.0 mL LB medium at 150 rpm and 37 °C for 12 h. 100 µL of this culture (OD_600_) was transferred to wells of a sterile 96-well culture dish followed by the addition of different concentrations of Au NPs. The pure Au NPs solution was used as a positive control, while sterile water was used as the negative control. The experiments were performed in triplicate. After covering, the 96-well culture dish was placed in an incubator at 37 °C for 12 h without agitation. The same procedure was adapted for fungal strains and data was recorded. After incubation, the OD_600_ was analyzed on an ELISA reader. A microbial growth curve was obtained by plotting the concentration of Au NPs (µg/mL) against OD_600_. The concentration of Au NPs that decreased the 95% microbial cell growth was considered as MIC value.

### In vivo wound healing activity of Au NPs

The effect of Au NPs in wound healing was investigated by topical application of Au NPs to the wounds generated in mice. Two symmetrical wounds were generated on both sides of the skin of each mouse by biopsy punch. The suspended Au NPs were topically applied to the wound followed by covering the wounds with 3 M Tegaderm™ film (6 cm × 7 cm). The wound healing process was investigated for 5 days. On the sixth day of wound generation, the mice were dissected, the mouse skin was excised around the wounds, and was stored in 1× PBS buffer at – 70 °C for further use.

The change in expression of wound healing biomarkers was investigated by quantitative PCR analysis. A simple method based on TRIzol reagent was employed to extract the total RNA from the tissues excised from wounds. The RNA was quantified by NanoDropTM spectrophotometer and cDNA was synthesized by cDNA synthesis kit (Thermo Fischer Scientific, USA) using 1 µg of RNA. Commercially synthesized oligonucleotide primers were used to amplify the cDNA. The CFX96 Real-time PCR Detection System (Bio-Rad Laboratories, Hercules, CA, USA) was used to quantify the expression of biomarkers in the tissues. The expression of the GAPDH gene was used as an internal control to normalize the expression of target genes. The reaction mixture was prepared according to instructions given in the kit manual. Analysis of the data was performed on CFX Manager Software (Bio-Rad, Hercules, CA, USA).

### Statistical analysis

All the statistical analyses were performed using SPSS software by International Business Machines Corporation (USA). The means of control and test groups for the antimicrobial and wound healing were compared using one-way ANOVA and values were expressed as mean ± standard deviation. A p < 0.05 was considered statistically significant.

### Ethics statement

All methods were accomplished consistently with the pertinent guidelines and regulations, and the experiments with animal models were in line with standard guidelines. The experimental design for experimental animal use was approved by the local Biosafety and Bioethics Committee at the University of the Punjab, Lahore, Pakistan. We hereby confirm that the study is reported in accordance with ARRIVE guidelines (https://arriveguidelines.org). The principles of replacement, reduction, and refinement (the 3Rs) were followed.

## Results and discussion

### Synthesis of Au nanoparticles

**Figure 1:**
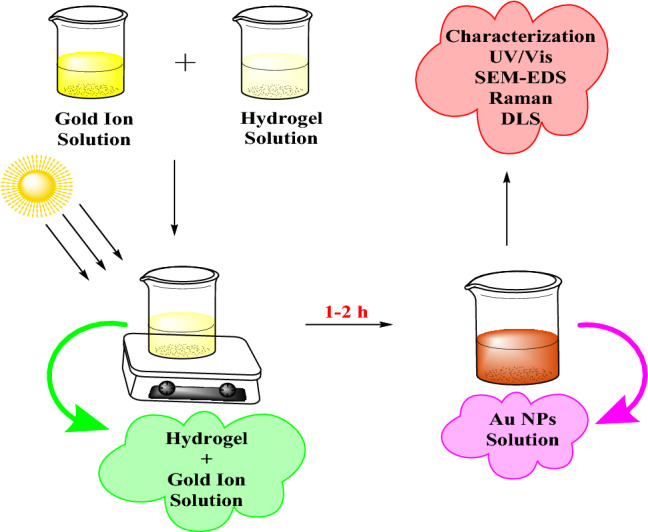
Schematic representation of the sunlight-assisted synthesis of Au NPs.

Light assisted green method was employed for the synthesis of Au NPs. The reaction proceeded by nucleation of gold atoms in the presence of hydrogel extracted from *C. oblonga* seeds and resulted in the formation of NPs. We propose that at the initial stages of the reaction, [Au(hydrogel)]^+^ complex is formed that is further reduced to a [Au(hydrogel)] complex. The functional groups present on the hydrogel stabilize the complex. The entire process is viewable through the colour variation from yellowish to reddish-brown (Fig. 1). This colour change originates from typical surface plasmon indicating the formation of Au NPs.

### Characterization of Au NPs

#### UV–Vis spectrophotometry

**Figure 2:**
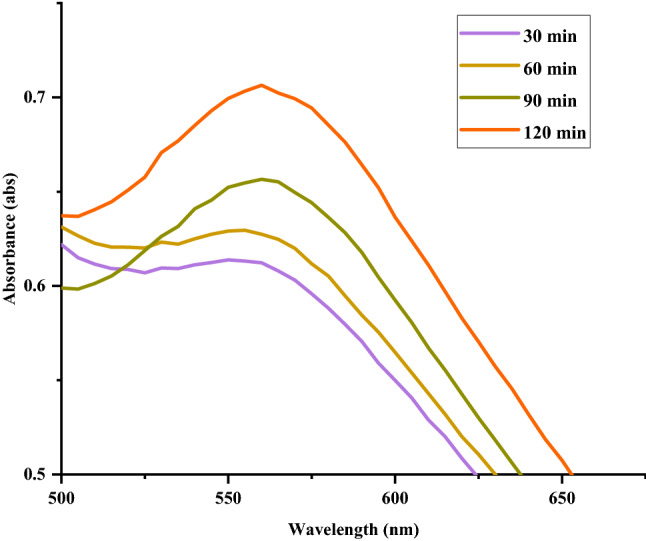
UV–Vis spectra show the changes in absorption of Au NPs at different times during synthesis.

The optical properties of nanoparticles mainly depend upon morphological characteristics such as size, shape, and surface. The UV–Vis spectra can reveal the distribution of size and shape of the nanoparticles. The UV–Vis analysis can also help to investigate the concentration and aggregate formation of the nanoparticles. In our synthetic procedure, the hydrogel (10 mL) was added to the gold precursor (10 mL: 5, 10, 15, 20, 25 mmol) in a beaker at room temperature. The reduction of Au ions to Au NPs causes a colour change from yellow to reddish-brown upon exposure to sunlight. These transitions are relevant to elapsed time and size of Au NPs that are interpreted by the colour variation. The formation of Au NPs was further investigated by collecting electronic absorption spectra at various stages of synthesis. The UV–Vis spectra of Au NPs for optimized concentration (10 mmol) as a function of reaction time shown in Fig. 2 displays a characteristic absorption corresponding to LSPR of Au NPs consistent with the previous report on Au NPs prepared using natural polymers^5^. The graduate increase in the absorption intensity with time indicates the formation of Au NPs.

#### Raman and FTIR spectroscopy

**Figure 3:**
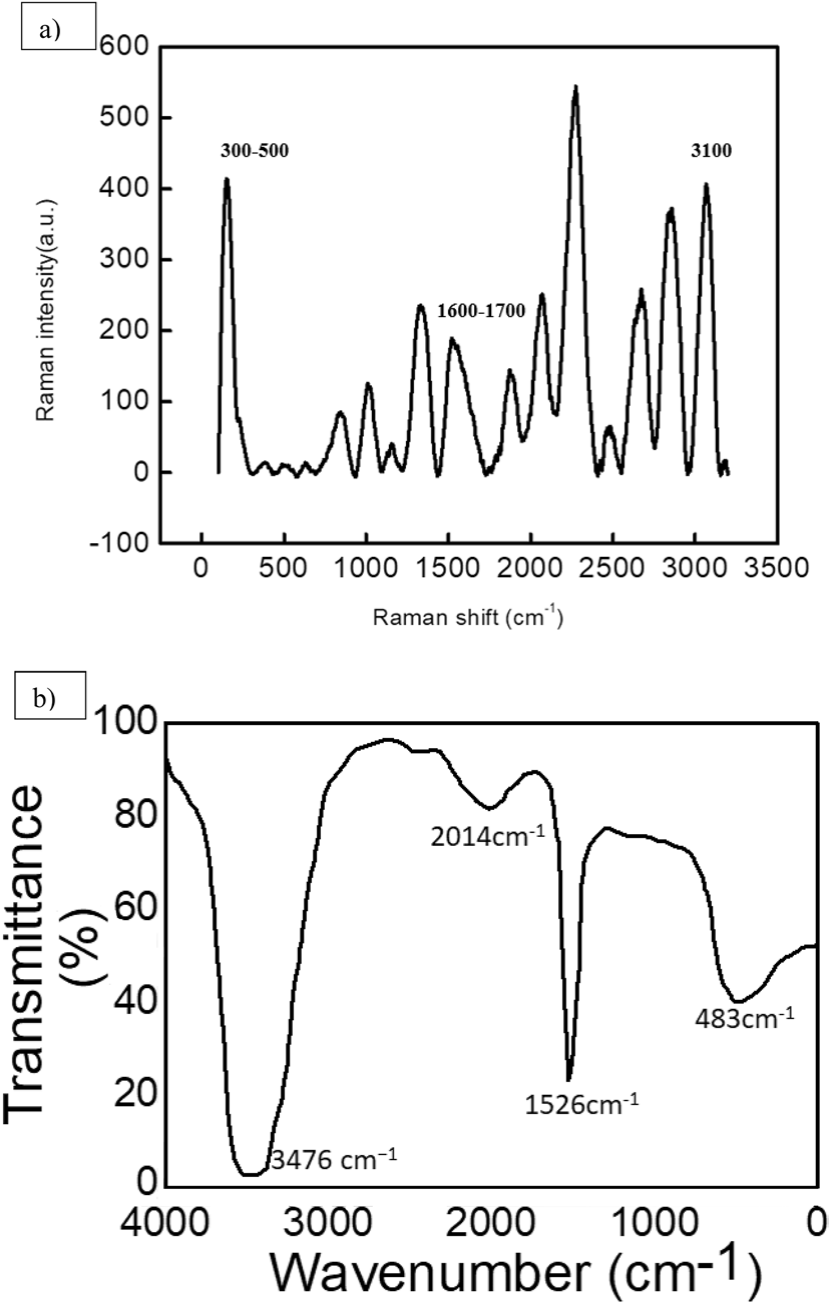
(**a**) Raman and (**b**) FTIR Spectrum for hydrogel-capped Au NPs.

The functional groups present in hydrogel stabilize the Au NPs. Raman spectroscopy can measure the vibrational fingerprint and identifies such functional groups. The formation of Au NPs and functional groups of hydrogels bound to Au NPs were investigated by Raman spectroscopy as shown in Fig. 3a. The analysis of Raman spectrum of as-prepared Au NPs showed a characteristic peak at 300–500 cm^−1^ indicating the successful formation of Au NPs with homogenous size. The peak at 1600–1700 cm^−1^ corresponds to carbonyl groups in the hydrogel while the peak at 3100 cm^−1^ corresponds to hydroxyl groups of the hydrogel. These results indicate that hydrogel functional groups are successfully bonded and stabilize the Au NPs. The results are consistent with the previous report where streptavidin on Au NPs-coated polystyrene beads was evaluated by Raman spectroscopy^29^. FTIR spectra had shown different stretching and bending vibrational modes at 483, 1526, 2014, and 3476 cm^−1^ of Au NPs extracted from C. oblonga plant (Fig. 3b). Stretching vibrational mode of C=C has been observed at 1526 cm^−1^. Weak overtone signal has been observed at 2014 cm^−1^ confirming the presence of aromatic components at the surface of NPs. Similarly, –OH stretching vibrations have also been observed at 3416 cm^−1^ due to the presence of terpene and fatty acid. Additionally, C–Br stretching vibrational mode was observed at 483 cm^−1^, thus indicating the presence of 4-bromo-1-naphtalenamine.

#### TEM analysis

**Figure 4:**
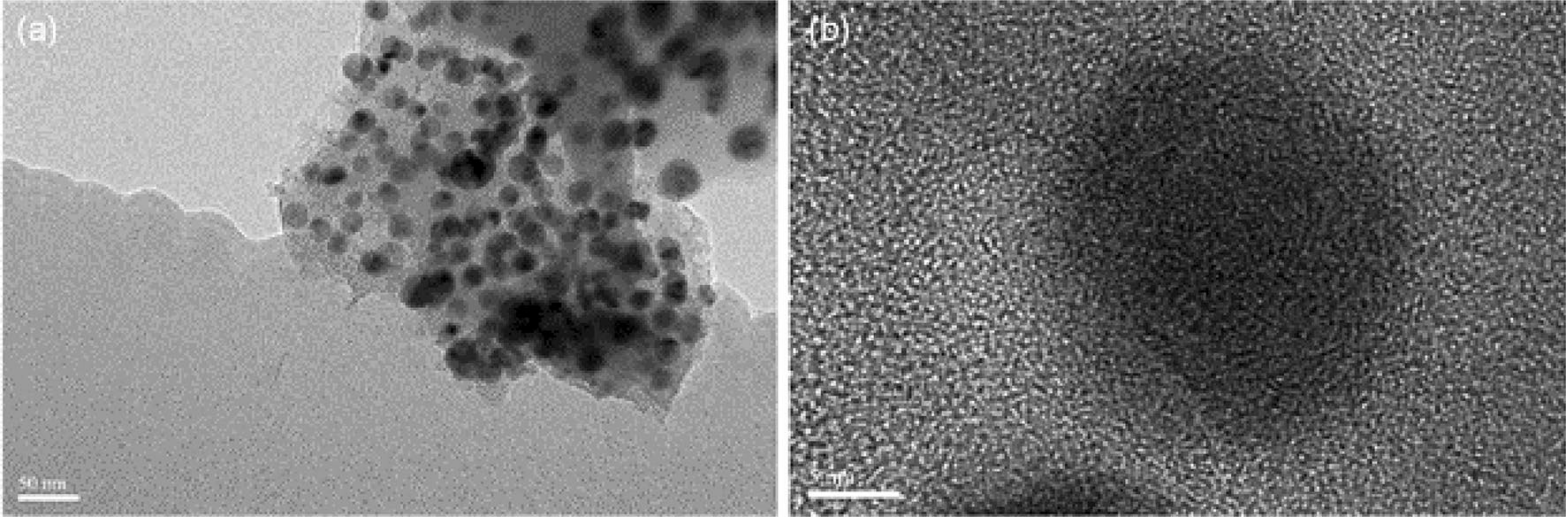
TEM images of Au NPs reveal the morphology and particle size.

TEM analysis was used to investigate the size and shape of the synthesized NPs. In the present study, the TEM images of Au NPs revealed the size of Au NPs in the range of 20 to 30 nm synthesized from the optimized concentration of precursor. The results are in good agreement with previous studies that report the particle size range of 50–100 nm for Au NPs synthesized in the presence of marine microorganisms^30^. TEM images indicated that most of the nanoparticles formed are spherical shaped (Fig. 4a,b).

#### Particle size analysis

**Figure 5:**
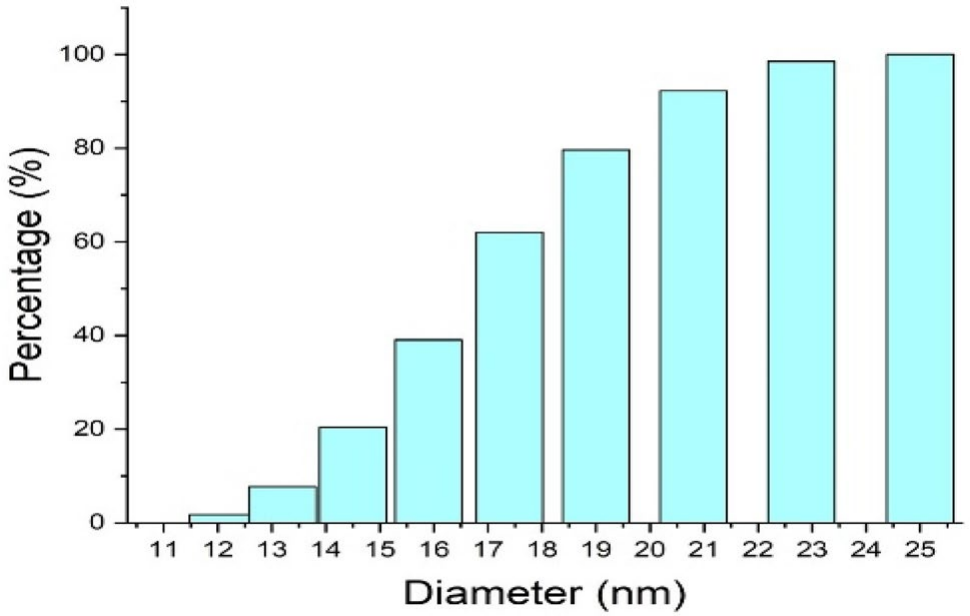
Graph showing the particle size distribution of synthesized Au NPs using particle size analyzer.

The particle size distribution is a significant feature of NPs. The dynamic light scattering technique can be employed to investigate the size distribution of nanoparticles^31^. The hydrogel-mediated Au NPs show different size distributions as indicated in Fig. 5. It is supposed that there are many particles with a size distribution of 25 nm. The results of the present study are supported by several previous studies summarized in Table 1.

**Table 1:** Comparison of size and shape of Au NPs with previous studies.

NPs	Particle shape	Particle size	References
Au NPs	Spherical shaped	25 nm	This study
Au NPs	Spherical	32 nm	^5^
Au-Se NPs	Spherical core/shell	25 nm	^7^
Ag NPs, Au NPs and Ag-Au NPs	Spherical	< 40 nm	^8^

#### XRD studies

**Figure 6:**
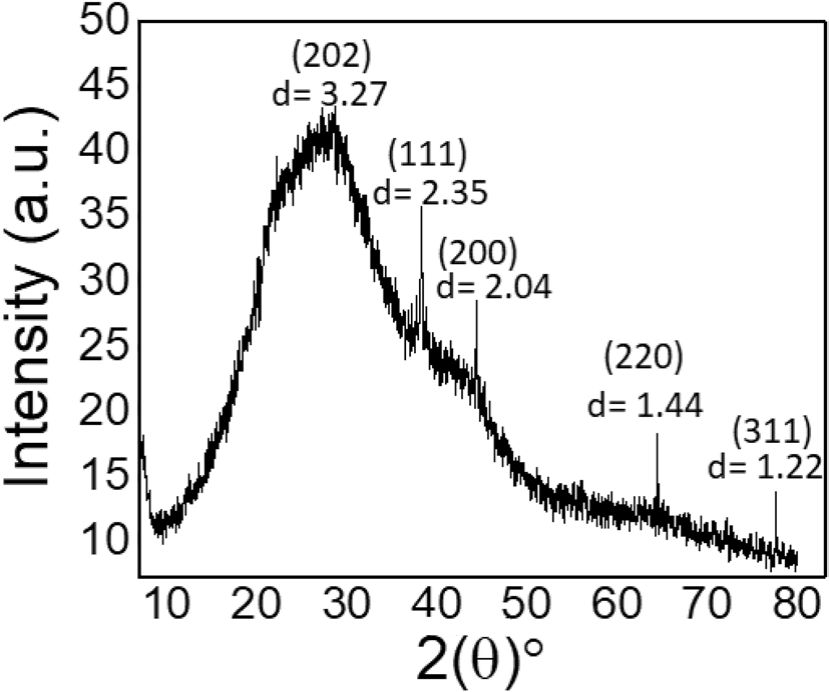
XRD pattern of Au NPs confirming the cubic structure.

Hydrogel-based Au NPs particles exhibited specific XRD patterns when recorded in the scanning range of 5 to 80° (Fig. 6). The diffraction peaks at 2θ of 38.28, 44.34, 64.46, and 77.58° corresponding to (111), (200), (220), and (311) crystal planes, respectively confirmed the presence of Au NPs with cubic symmetry as is evident from 04-0784 file of the JCPDS database. The d-spacing values with respect to planes are also shown in Fig. 6. The broad peak before 38° is due to the amorphous nature of the hydrogel.

#### Microbicidal activity of Au NPs

**Figure 7:**
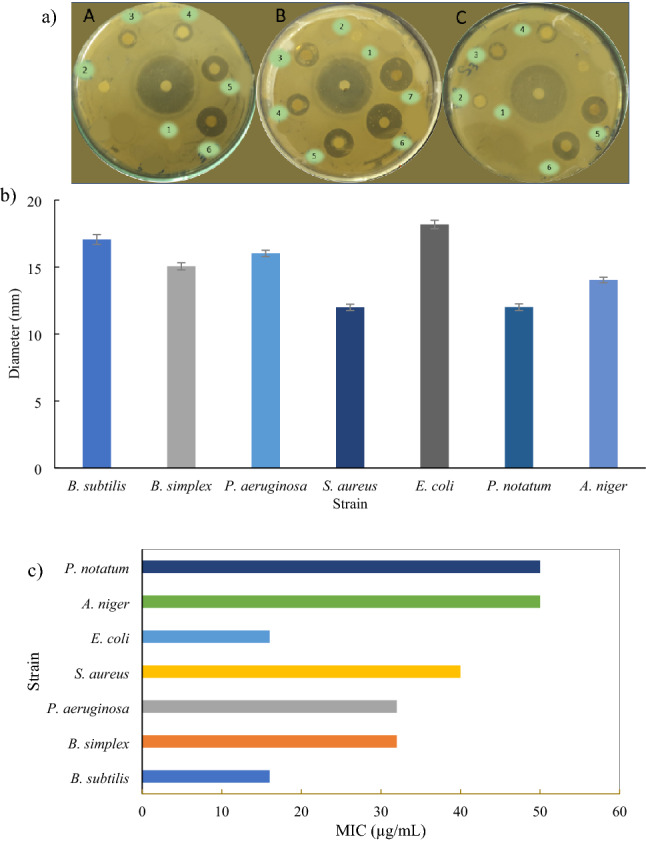
The anti-microbial potential of Au NPs: (**a**) Zones of inhibition of model bacterial strains (A) *B. simplex*, (B) *E. coli*, (C) *S. aureus*; 1: Ampicillin; 2–7: 10–50 µL/mL Au NPs. (**b**) Graph showing maximum diameter for zones of inhibition of various microbial strains. The error bars show the standard deviation. (**c**) Graph showing MIC values of various microbial strains.

The fungal and bacterial growth is greatly constrained by the synthesized Au NPs. Initially, the formation of the zone of inhibition was investigated. Au NPs inhibited the growth of microorganisms and clear zones of inhibition were formed with *B. simplex, E. coli,* and *S. aureus* (Fig. 7a). The as-prepared Au NPs inhibited the growth of *B. simplex*, *B. subtilis*, *P. aeruginosa*, *E. coli*, and *S. aureus* with inhibitions zones of 15, 17, 16, 18, and 12 mm, respectively on LB agar plates in 24 h. The zones of inhibition for fungal strains such as *P. notatum* and *A. niger* were found to be 12 and 14 mm for 10 mm Au NPs solution (Fig. 7b). However, no antimicrobial activity was seen in the suspension of hydrogel in water. All the assays were performed in triplicates, and the average values are being reported. The MIC values of Au NPs against different bacterial strains i.e., *B. simplex, B. subtilis, P. aeruginosa, E. coli,* and *S. aureus* were 16, 32, 32, 16, and 40 mg mL^−1^ respectively, and 50 mg mL^−1^ for both fungal strains (*A. niger* and *P. notatum*) (Fig. 7c).

### Phenotypic analysis of wound healing

**Figure 8:**
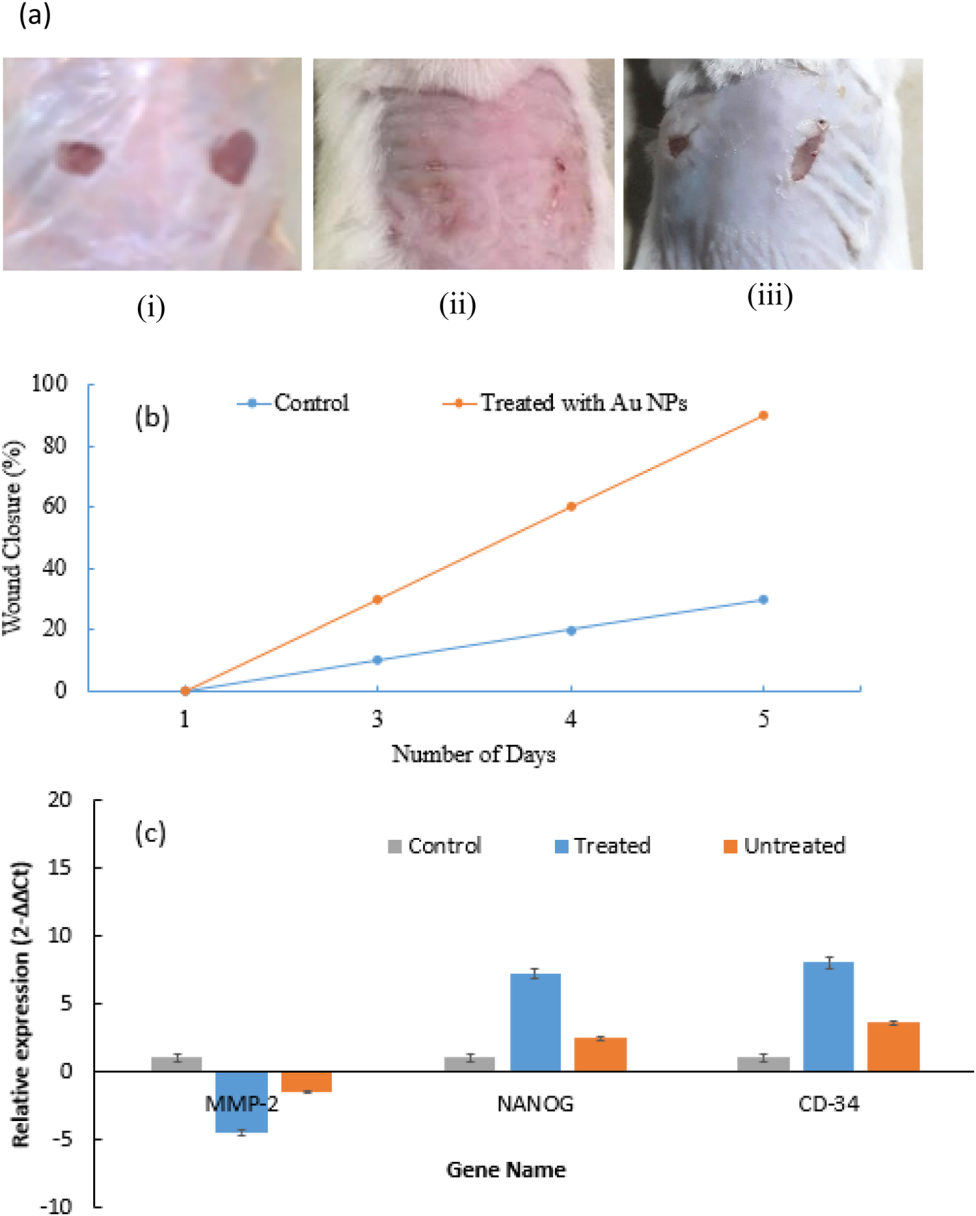
Au NPs accelerated wound healing in mice. (**a**) (i) mice on wound generation day, (ii) healing of wounds in mice treated with nanoparticles and (iii) untreated mice. (**b**) The graph shows % wound closure in control and the mice treated with Au NPs over time. (**c**) Relative expression of biomarkers of wound healing (MMP-2, NANOG & CD-34) in the skin tissues of treated and untreated mice groups. The error bars show the standard deviation.

Au NPs promoted wound healing by either inhibiting the growth of bacteria and fungi or stimulating the release of wound healing factors. Recently Au NPs have been shown to increase the adhesion properties of human fibroblast cells^7^. The wound healing process in mice was observed phenotypically as shown in Fig. 8a. The diameter of the wound was measured in mice at the start of the experiment and after 3, 4, and 5 days following the wound generation. Percentage wound closure was also calculated by the following formula:

$$\% \; \text{Wound} \; \text{closure}=\frac{\text{Diameter} \; \text{of} \; \text{original} \; \text{wound}-\text{Diameter} \; \text{ of} \; \text{the} \; \text{healed} \; \text{wound}}{\text{Diameter} \; \text{original} \; \text{wound}}\times 100$$

It was observed that about 90% of a wound upon treatment with nanoparticles was healed on the 5th day of treatment as compared to wound in control mice (p < 0.000) (Fig. 8a, Table 2). The percent wound closure in mice with respect to time is shown in Fig. 8b.

**Table 2:** Wound closure in mice after 3, 4, and 5 days.

Group	Diameter of original wound in mm	Diameter of actual wound in mm After 3 days	Diameter of actual wound in mm after 4 days	Diameter of actual wound in mm after 5 days
Control^a^	5.00	4.50	4.00	3.00
SD	± 0.17	± 0.1	± 0.1	± 0.26
N	3	3	3	3
Treated^a^	5.03	3.50	2.03	0.50
SD	± 0.21	± 0.26	± 0.21	± 0.2
N	3	3	3	3

^a^Mean value.

#### Quantitative analysis of wound healing biomarkers

Transcription factor NANOG maintains pluripotency and promotes wound healing^32^. Increased level of transmembrane adhesion protein CD34 is typically associated with enhanced wound healing^33^. The change in the expression of NANOG, CD34, and MMP-2 genes related to wound healing was measured by quantitative real-time PCR measurements. According to our results, the expression of NANOG and CD-4 was significantly increased in the wound tissues treated with nanoparticles as compared to the untreated. The expression of MMP-2 was also decreased in treated wound tissues as compared to untreated wound tissues in the mice (Fig. 8c).

## Conclusions

The present study reports a new sunlight-assisted green method to synthesize Au NPs using hydrogel extracted from seeds of *C. oblonga*. Hydrogel extracted from the seeds of *C. oblonga* is a novel reducing and stabilizing agent for Au salts that has not been employed previously. Cubic and rectangular-shaped Au NPs with an average edge length of 74 ± 4.57 nm were synthesized. Raman spectroscopy confirmed the successful capping of Au NPs with carboxyl and hydroxyl groups of the hydrogel. The results reported here revealed the significant antimicrobial potential of Au NPs. We found that these NPs enhanced wound healing by promoting the population of pluripotent cells and transmembrane proteins. Owing to the medicinal properties of *C. oblonga* plant and convincing antimicrobial properties, these NPs can play a vital role in biomedical applications for imaging, controlled drug delivery, diagnosis, cancer treatment, and many others.
